# Concomitant treatment of sustained ventricular tachycardia and hypertrophic cardiomyopathy with transcoronary ethanol ablation: a case report

**DOI:** 10.1093/ehjcr/ytad632

**Published:** 2023-12-23

**Authors:** Eric T Xia, Kevin Lee, Iva Minga, Jose Nazari, Mark D Metzl

**Affiliations:** Department of Cardiology, University of Chicago Northshore University Health Systems, 2650 Ridge Avenue, Evanston, IL 60201, USA; Department of Cardiology, University of Chicago Northshore University Health Systems, 2650 Ridge Avenue, Evanston, IL 60201, USA; Department of Cardiology, University of Chicago Northshore University Health Systems, 2650 Ridge Avenue, Evanston, IL 60201, USA; Department of Cardiology, University of Chicago Northshore University Health Systems, 2650 Ridge Avenue, Evanston, IL 60201, USA; Department of Cardiology, University of Chicago Northshore University Health Systems, 2650 Ridge Avenue, Evanston, IL 60201, USA

**Keywords:** Transcoronary ethanol ablation, Ventricular tachycardia, Cardiac magnetic resonance imaging, Percutaneous coronary intervention, Hypertrophic cardiomyopathy, Case report

## Abstract

**Background:**

The recommended treatment for recurrent ventricular tachycardia in patients with hypertrophic cardiomyopathy that is not amenable to defibrillator implantation due to shock burden is radiofrequency ablation. In patients with deeply intramural foci of ventricular tachycardia, traditional unipolar ablation has a lower probability of success.

**Case summary:**

A 66-year-old Caucasian man was admitted with ventricular tachycardia, which recurred despite antiarrhythmic drugs. On cardiac magnetic resonance imaging, he was discovered to have septal hypertrophic cardiomyopathy, which was not significant on echocardiogram. The focus of ventricular tachycardia was suspected to be buried deeply within the hypertrophic segment as localized by late gadolinium enhancement. The patient underwent transcoronary ethanol ablation, which abated the ventricular tachycardia while also completely decreasing his invasively measured left ventricular outflow tract obstruction gradient from 45 to 17 mmHg.

**Discussion:**

Transcoronary ethanol ablation may be successfully applied to simultaneously treat ventricular arrhythmia superimposed within a segment of hypertrophic cardiomyopathy. Further data are needed to evaluate long-term success of this strategy vs. traditional radiofrequency ablation.

**Learning points**

- First-line treatment for recurrent ventricular tachycardia is implanted cardiac defibrillator followed by radiofrequency ablation by an electrophysiologist.
- In cases where there is a concurrent indication for septal ethanol ablation for obstructive hypertrophic cardiomyopathy, transcoronary ethanol ablation may be selected first to treat septally originating ventricular tachycardia.

## Introduction

The guideline-recommended treatment for medication refractory monomorphic ventricular tachycardia (VT) in patients with concomitant hypertrophic cardiomyopathy (HCM) is radiofrequency ablation (RFA).^1^ In some cases, the VT substrate is deep within the hypertrophied segment, which makes traditional RFA difficult. Several approaches have been described that can be applied to treat VT originating within hypertrophic segments. The two most widely utilized approaches are simultaneous epicardial/endocardial RFA and RFA using 0.45% normal saline as high impedance irrigation solution.^2,3^ Bipolar RFA aims to create deeper lesions for a given width but has a concerning, although improving, safety profile.^4^ Lastly, infusion needle RFA involves piercing an extendable needle to deliver energy directly to the deep segment but showed limited success in a pilot trial.^5^ Herein, we present a patient with refractory VT suspected to originate from deep within a hypertrophic septum and the utilization of transcoronary ethanol ablation (TCEA) to treat VT and HCM simultaneously.

## Summary figure

**Table:** 

At emergency room presentation	Symptomatic VT initially controlled with amiodarone and metoprolol.
Intensive care unit admission	Multiple recurrences of VT, ultimately controlled with procainamide.
Day 1	Cardiac catheterization with normal coronaries and echocardiogram with 14 mm maximum septal diameter.
Day 2	Cardiac magnetic resonance imaging (cMRI) revealing 18 mm maximum septal diameter with septal late gadolinium enhancement (LGE).
	Combined with electrocardiogram (ECG), localized VT focus to hypertrophic septum.
Day 5	Transcoronary ethanol ablation, reducing left ventricular outlet obstruction gradient from 45 to 17 mmHg.
3 months later	Repeat imaging confirming post procedure findings. No defibrillator shocks.
4 months later	Five runs of VT broken by pacing. Started on amiodarone.
1 year later	Stable on 100 mg daily amiodarone. No plans for further ablations.

## Case

**Figure 1:**
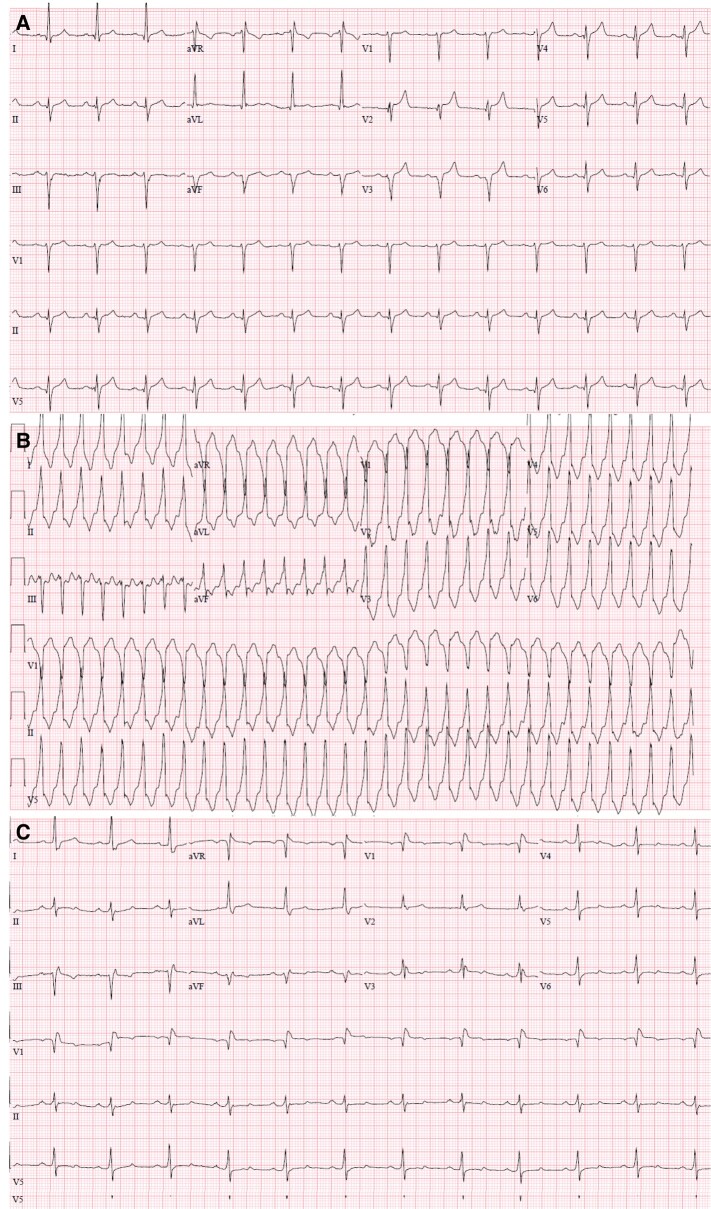
(*A*) Baseline electrocardiogram. (*B*) Presenting electrocardiogram showing monomorphic ventricular tachycardia at presentation with normal axis and early transition of QRS complexes in lead V2. (*C*) Post ablation electrocardiogram with right bundle branch abnormality.

A 66-year-old Caucasian male with history of coronary artery disease (CAD) complicated by unstable angina status post three-vessel stenting, insulin-dependent type 2 diabetes, and essential hypertension presented to the emergency department with palpitations, presyncope, and intermittent chest pain for 4 days. Blood pressure was 108/85 mmHg, respiratory rate was 20/min, and physical examination was unremarkable except for tachycardia. Blood count and metabolic profile were both unremarkable. High-sensitivity troponin peaked at 156. Electrocardiogram revealed monomorphic VT with normal axis and early transition of QRS complexes in lead V2 (*Figure 1A* and *B*). Ventricular tachycardia was initially aborted with intravenous amiodarone bolus followed by lidocaine infusion plus as-needed intravenous metoprolol. However, the patient experienced three episodes of sustained VT on the first 24 h of admission, qualifying a diagnosis of VT storm.

**Figure 2:**
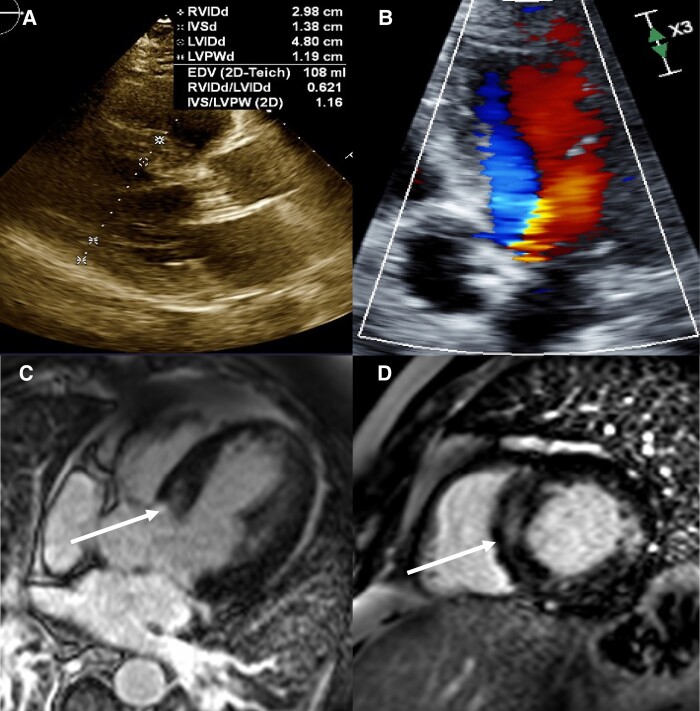
(*A*) Parasternal long transthoracic echocardiogram view likely underestimating intraventricular septal diameter. (*B*) Apical four-chamber transthoracic echocardiogram Doppler of left ventricular outflow tract obstruction. (*C*) Four-chamber cardiac magnetic resonance imaging view demonstrating intramyocardial late gadolinium enhancement (arrow). (*D*) Basal short-axis cardiac magnetic resonance imaging view of late gadolinium enhancement (arrow).

Suspected aetiologies included ischaemic VT, scar-mediated VT, and idiopathic left ventricular outflow tract (LVOT) VT originating from the basal septum given the QRS morphology on the ECG. Transthoracic echocardiogram (TTE) revealed left ventricular ejection fraction (LVEF) of 55% and an interventricular septal diameter (IVSd) of 14 mm, which was unchanged from prior TTEs. Peak LVOT gradient was measured at 25 mmHg (*Figure 2A* and *B*). A coronary angiogram revealed patent stents and non-obstructive CAD. Cardiac magnetic resonance imaging revealed IVSd measuring up to 18 mm in the mid-basal septum with mid-myocardial LGE within the hypertrophic segment corresponding to likely fibrosis (*Figure 2C* and *D*; see Supplementary material online). No LGE was observed in the territories of prior coronary intervention. Prior literature correlates LGE with VT foci.^6^ The QRS morphology also suggested septal origin.^7^ Given the diagnostic clarity achieved with imaging, an electrophysiology mapping study was bypassed.^8^

**Figure 3:**
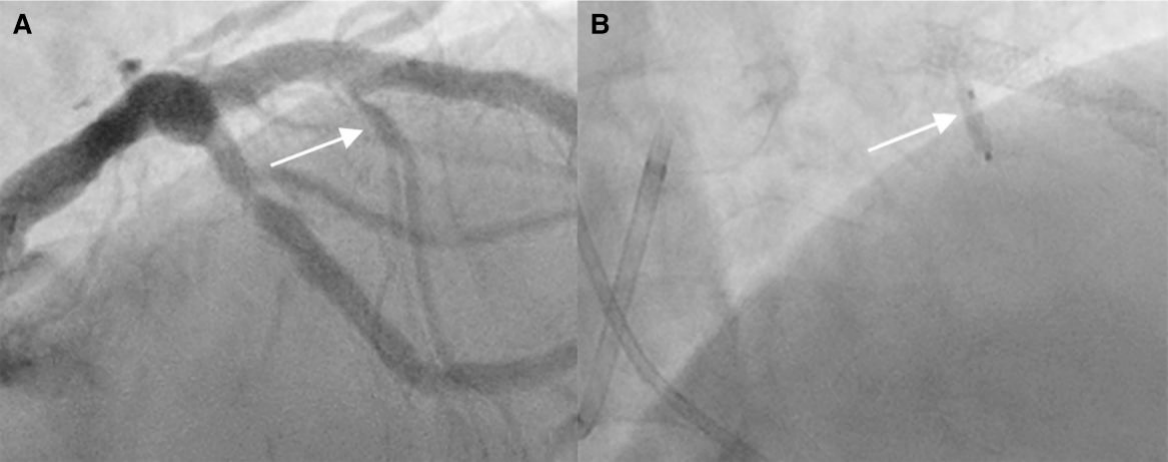
(*A*) View of the first septal branch prior to ballooning and ethanol injection and (*B*) 2.0 × 8 mm over the wire balloon in the first septal branch.

Considering the significant burden of recurrent VT despite antiarrhythmic drug therapy, RFA was recommended prior to Implantable Cardiac Defibrilator (ICD) placement. All ECG and imaging data thus far suggested that fibrosis within the hypertrophic septum should be the target of ablation. However, because the stripe of LGE was so central, it was believed that traditional RFA would have a low chance of completely ablating the segment. Several strategies were considered including simultaneous epicardial–endocardial ablation, bipolar ablation from the left and right ventricles, and surgical myectomy. Review of existing literature did not support one approach as superior.^2,9,10,11^ During the original echocardiogram, the 25 mmHg LVOT gradient was misinterpreted as aortic stenosis and no gradient under Valsalva was measured. Given the 18 mm IVSd, it was suspected that the true gradient was significantly higher. Coronary angiogram showed favourable anatomy for successful septal branch ablation (*Figure 3A*). Therefore, TCEA was chosen to ablate the focus of VT while also potentially ameliorating the patient’s LVOT obstruction.

Dual arterial access was established for simultaneous left ventricle and aortic pressure measurement. Resting post-premature ventricular contraction (PVC) gradient across the LVOT was 45 mmHg. A balloon was advanced into the first septal perforator branch (*Figure 3B*). With the balloon inflated, 1.5 mL of ethanol was injected into the first septal branch. Post-PVC gradient was reduced to 17 mmHg after ablation. A new right bundle branch abnormality was seen post ablation (*Figure 1C*). Repeat TTE revealed LVOT gradient of 15 mmHg, akinesis of the basal septum, and LVEF 55%. An ICD was then implanted, and the patient was discharged on oral metoprolol alone. At 4 months post-TCEA, the patient was noted to have five runs of VT aborted with antitachycardia pacing and amiodarone was initiated. At 1 year post-procedure, the patient had only one episode of VT in the preceding 6 months. He takes 100 mg of amiodarone daily, and there are no plans for further ablation.

## Discussion

In patients with sustained VT in the setting of HCM, evidence-based decision-making regarding the choice of procedure is challenging. Studies of RFA outcomes in this population are small and describe patients with a mixture of hypertrophy patterns, VT substrate locations, and CAD histories.^2,9,10,11^ For all patients, ICD placement is the most effective at reducing cardiac mortality.^1^ Medical therapy should be initiated concurrently, starting with rate control medications before progressing to antiarrhythmic drugs. This medication strategy mimics that used to treat VT in patients with history of CAD. However, no study has directly compared outcomes of different medical therapies in VT in the setting of HCM. Patients who continue to have breakthrough VT may be considered for invasive therapy. The only procedure with significant supporting data is RFA, which holds a IIb class recommendation in the 2022 ESC Guidelines for ventricular arrhythmias.^1^

In our patient, cMRI informed the suspected source of VT. Sakamoto *et al.*^6^ described the use of cMRI to localize VT in this population, performing contrast-enhanced cMRI in 50 consecutive patients with HCM. Of these participants, 18 patients with a history of VT underwent electrophysiology mapping and conduction testing. All 18 VT foci were within LGE-positive segments, which also exhibited electrical dysfunction consistent with hypertrophic scarring. With the added context of this patient’s ECG, these findings supported the belief that the VT focus was located in the stripe of LGE deep within the hypertrophic basal septum.

To date, only six studies with 68 total patients have charted VT RFA outcomes in patients with HCM. Meta-analysis reports that 84.5% of patients achieved acute procedural success. In follow-up, freedom from recurrent VT was 70.2% (95% confidence interval: 51.9–86.2%).^9^ While generally supporting favourable outcomes of RFA, it is difficult to apply these data to individual patients because of the previously noted variability in patient characteristics and follow-up times.^2,9,10,11^

The main novelty of this case is the choice to use TCEA as a first-line strategy. Conceptually, TCEA is attractive as it can potentially treat both HCM and VT simultaneously. However, septal TCEA can cause complete heart block with rates reported at 14%.^12^ Transcoronary ethanol ablation is routinely offered following failed RFA but is perceived as second-line or salvage therapy. As the current guidelines suggest, more data are needed to evaluate the relative efficacy of different ablation techniques in this patient population.

## Conclusion

Recurrent VT in patients with HCM is a treatment challenge. This case report describes the successful TCEA of a critically ill patient, which successfully relieved both recurrent VT and LVOT obstruction. This report is not a recommendation for TCEA to be attempted prior to RFA, although the available data indicate that this choice is reasonable. Significantly more data are needed to guide clinicians in their procedural choices when treating patients with VT in the setting of HCM.

## Supplementary Material

ytad632_Supplementary_Data

## Data Availability

No new data were generated or analysed in support of this research.

## Lead author biography

Dr Xia graduated from Marian University School of Osteopathic Medicine and is currently completing an internal medicine residency at the University of Chicago Northshore. He has a clinical interest in electrophysiology and advanced heart failure. He enjoys sports and spending time with his growing family.
